# Major phenolic compounds, antioxidant, antimicrobial, and cytotoxic activities of *Selinum carvifolia* (L.) collected from different altitudes in India

**DOI:** 10.3389/fnut.2023.1180225

**Published:** 2023-07-13

**Authors:** Ravi Prakash Srivastava, Sachin Kumar, Lav Singh, Mayank Madhukar, Nitesh Singh, Gauri Saxena, Shivaraman Pandey, Arpit Singh, Hari Prasad Devkota, Praveen C. Verma, Shatrughan Shiva, Sumira Malik, Sarvesh Rustagi

**Affiliations:** ^1^Department of Botany, University of Lucknow, Lucknow, Uttar Pradesh, India; ^2^Food, Drug and Chemical Toxicology Group, CSIR-Indian Institute of Toxicology Research (CSIR-IITR), Lucknow, Uttar Pradesh, India; ^3^Forest Training Institute, Ministry of Environment, Forest and Climate Change, Govt. of Uttar Pradesh, Kanpur, India; ^4^PG Department of Zoology, RD and DJ College, Munger University, Bihar, India; ^5^Faculty of Agricultural Sciences, Shree Guru Gobind Singh Tricentenary University, Gurugram, Haryana, India; ^6^Graduate School of Pharmaceutical Sciences, Kumamoto University, Kumamoto, Japan; ^7^Plant Molecular Biology and Genetic Engineering Laboratory, Council of Scientific and Industrial Research, National Botanical Research Institute (CSIR-NBRI), Lucknow, Uttar Pradesh, India; ^8^Amity Institute of Biotechnology, Amity University, Ranchi, Jharkhand, India; ^9^School of Applied and Life Sciences, Uttaranchal University, Dehradun, Uttarakhand, India

**Keywords:** antimicrobial, antioxidant, HPLC, MTT assay, pharmacology, *Selinum carvifolia*

## Abstract

Antibiotic resistance poses a serious threat to public health, raising the number of diseases in the community. Recent research has shown that plant-derived phenolic compounds have strong antimicrobial, antifungal, and cytotoxic properties against a variety of microorganisms and work as great antioxidants in such treatments. The goal of the current work is to evaluate the anticancerous, antibacterial, antifungal, antioxidant, and cytotoxicity activities in the extracts of the different plant parts (leaves, stems, and roots) of *S. carvifolia* (L.) L. This is a medicinally important plant and has been used for different kinds of diseases and ailments such as hysteria and seizures. The phenolic compounds from the different plant parts were analyzed using HPLC and the following were found to be present: chlorogenic acid, gallic acid, rutin, syringic acid, vanillic acid, cinnamic acid, caffeic acid, and protocatechuic acid. Gallic acid was found to have the highest concentration (13.93 mg/g), while chlorogenic acid (0.25 mg/g) had the lowest. The maximum TPC value, which ranged from 33.79 to 57.95 mg GAE/g dry extract weight, was found in the stem. Root extract with 9.4 mg RE/g had the greatest TFC level. In the leaf and stem extracts, the RSC ranged from 0.747 mg/mL to 0.734 mg/1 mL GE/g dry extract weight, respectively. The 2,2-diphenyl-1-picrylhydrazyl (DPPH) assay was used to measure *in vitro* antioxidant activity. In a concentration-dependent way, promising antioxidant activity was reported. Moreover, 3,5-dinitrosalicylic acid (DNSA) and the Folin–Ciocalteu phenol reagent technique were used to determine reducing sugar content and total phenolic content, respectively. Antibacterial activity against eight strains (MIC: 250–1,000 μg/mL) was analyzed, and the stem extract exhibited maximum activity. Antifungal activity was also assessed, and potent activity was reported especially in the extract obtained from the stem. Cytotoxicity was evaluated using an MTT assay in the A549 cell line, where different doses (0.0625, 0.125, 0.25, 0.5, and 1 mg/mL) of leaf, root, and stem extracts were used. Treatment with these extracts reduced the cell viability, indicating that *S. carvifolia* may possess anticancer potential, which can be of great therapeutic value.

## Introduction

A serious concern to the public’s health is the emergence of pathogenic bacteria and fungi that become resistant to synthetic antibacterial treatments over a period of time. This may result in an increased load of several antibiotic resistant bacteria and illnesses caused by them (1).

Most of the conventional antibiotics currently available for purchase have significant adverse effects on patients and have been responsible for the emergence of multiple drug resistance in pathogenic bacteria (2). Previous research has shown that bactericidal antibiotics such as quinolones and aminoglycosides have negative side effects, while lactams cause mitochondrial dysfunction and excessive production of reactive oxygen species (ROS) in mammalian cells, resulting in oxidative damage to DNA, proteins, and membrane lipids (3). Free radicals have also been linked to a number of diseases, including ischemic heart disease, diabetes, atherosclerosis, cancer, inflammation, and aging (4).

The defense against free radicals and harmful microbes is greatly aided by secondary plant metabolites such as phenols, flavonoids, and terpenoids (5). In order to tackle these resistant infections while avoiding or limiting the side effects associated with the consumption of synthetic antibiotics, there has been increasing interest in the identification of novel natural antimicrobial agents (6). As a result, combining antioxidant therapy with antibiotic medication appears to be a strategy to reduce or avoid these side effects. Plant-derived medicines cause few side effects, and they have a long history of use in folk medicine for the treatment of infectious diseases and oxidative stress conditions (7, 8).

The use of antioxidant and antibacterial drugs together helps to boost their antioxidant and antibacterial capabilities. It has also been observed that phenols, flavonoids, and terpenoids can increase the sensitivity of various bacteria to particular antibiotics (9–11).

The antibacterial and antioxidant properties of natural compounds from higher plants may prove to be a new source with a novel mechanism of action (12). As a result, there are three different levels of interaction at play: interaction with the outside cellular components, engagement with the cytoplasmic membrane, and interaction with cytoplasmic components. To exercise their antibacterial effects, natural compounds might interact with bacterial cells on one level or all three levels.

They may find innovative active principles to circumvent resistance mechanisms in multidrug-resistant microbes as a result of their thorough and systematic screening (13).

Many medicinally important plants can be found in the high altitude, alpine, and sub-alpine regions of Uttarakhand, Himachal Pradesh, and the surrounding areas of the Western Himalayas in India. The richness of medicinal plants in the Himalayas reflects its distinct geological, topographical, ecological, climatic, and physiographic position (14, 15). These plants have ethnobotanical significance and are often utilized in traditional medicine (16, 17). Medicinal plants are known to contain specific bioactive compounds that can inhibit microbial growth in the environment; therefore, they play a significant role in the creation of effective therapeutic treatments (18–20). Pharmacological industries have created a large number of antibiotics (21–23). Researchers have been investigating the antibacterial activity of medicinal plants as a result of the acceptance of traditional medicine as an alternative form of healthcare and the development of microbial resistance to current antibiotics (24–26).

Recent research has revealed that the antioxidant activity of plant products is mostly due to the presence of phenolic components including flavonoids, phenolic acids, tannins, and other phenolic compounds (27–29). Studies report that excessive free radicals are responsible for various pathological conditions such as asthma, arthritis, inflammation, neuro-degeneration, Parkinson's disease, and diabetes. Natural antioxidants have become the focus of a multitude of studies aimed at identifying sources of potentially safe, effective, and low-cost antioxidants (30, 31). Herbal medications that include free radical scavengers have been shown to have therapeutic value (32). To avoid the oxidation of the vulnerable substrate, plants create an astonishing array of antioxidant molecules such as carotenoids, flavonoids, ascorbic acid, and others. Antioxidants are commonly used in the food industry to prevent lipid peroxidation (33, 34). Plants include different iso-quinoline alkaloids that have antibacterial, anticancer, anti-inflammatory, adrenolytic, sympatholytic, and anti-acetylcholine esterase effects, according to chemical and pharmacological research (35, 36). The most efficient method for quantifying phenolic and flavonoid chemicals is HPLC analysis (37).

*Selinum carvifolia* plants have shown useful therapeutic effects, which are mostly dependent on the presence of phenols, flavonoids, and terpenoids. This is a perennial herb native to the high altitudes of Uttarakhand, Himachal Pradesh, and the neighboring regions of Indian Western Himalayas (38). This important species grows in humus-rich mountainous regions of the Himalayas between 2,200–4,000 m in states of Himachal Pradesh, Uttarakhand, and Sikkim in India and in neighboring countries such as Nepal and China (39). In India, the genus *Selinum*, also known as Bhutkeshi, is used as an insecticide, a nervine sedative with anti-spasmodic stimulating effects, and in the cure of constipation, menstruation, and digestion, among other things (40). A paste made from the leaves of *S. carvifolia* has been ethno-medically used for wound healing for centuries. The smoke produced from burning the roots of *S. carvifolia* is reported to repel insects, and the root decoction contains antimicrobial properties that can be used to cure coughs, colds, fevers, and body problems (41–43). However, due to their indiscriminate usage, the plants now need to be grown *in vitro* for their cultivation and preservation (44).

Previous studies on *Selinum* mainly focused on the volatile constituents of the different plant parts (45–49), but there are hardly any detailed reports on the non-volatile constituents in the leaves, stems, and roots of *Selinum carvifolium*. Recently, two novel compounds, bhutkesoside A (1) and bhutkesoside B, and 10 known compounds from the roots of the same plant have been reported from the leaves of *Ligusticopsis wallichianum* (50). The present study, therefore, aimed at quantifying and identifying the phytochemical constituents of the leaves, stems, and roots of *S. carvifolia* collected from three different altitudes (2,150, 2,593, and 3,178 m) in the Chopta region of Uttarakhand, India, and probing their antioxidant activity, reducing sugar capacity (RSC), and antibacterial and cytotoxicity properties in order to establish the therapeutic potential of this plant.

## Materials and methods

### Collection of plant material

The samples were collected in September 2018 from three different altitudes: 2,150 m (latitude 22°21′12.4″N75°12′01.4″E), 2,593 m (latitude 25°23′14.9”N75°11′01.3″E), and 3,178 m (latitude 28°24′25.9″N 75°10′57.6″E). Five to six whole plants were collected from each altitude in the Chopta region of Uttarakhand, India. A GPS positioning system was used for geographic coordinates, and the elevation information of each measurement point was recorded. The plants were identified by Dr. L. B. Chaudhary, Principal Scientist, Herbarium (Angiosperm Taxonomy), National Botanical Research Institute, Lucknow, India, and the samples were deposited in the NBRI repository as voucher specimen LWG 105543.

### HPLC

#### Analysis sample preparation

Ten grams of dry weight of raw material (roots, leaves, and stems) were powdered and poured into a conical flask. One hundred milliliters of 70% MeOH was added to it, and the extract thus formed was left for 48 h. Fractionation was performed in different solvents in the order of hexane (Hx), petroleum ether (PTE), and chloroform (Chl), as per the extraction methods of Abubakar and Haque (51). The residue was returned to the conical flask after the extracts from various plant sections were filtered through Whatman filter paper No.1 into a 250 mL volumetric flask. The extraction technique was performed two more times, with 70% methanol used to make up the difference in volume. Before the HPLC injection samples could be analyzed using a standardized HPLC procedure, each sample solution was filtered through a 0.2 μm membrane filter into a HPLC sample vial.

#### Instrumentation and conditions

The principal ingredients of all *S. carvifolia* extracts were analyzed by HPLC (Shimadzu, Japan) with PDA SPD M 20. A 20 μL sample loop was included with the LC-20 AD dual pump system and SIL-20 AC auto-injector (with cooler). Compounds were separated on a Shimadzu RP-C18 column with an internal diameter of 250 × 4.6 mm and a pore size of 5 m, which was protected by a guard column with the same packing. Subsequent elution of the column with hexane and a 2% ethyl acetate/hexane mixture yielded 100 mg of pure compounds infraction. The crude extract of leaf, stem, and roots was fractionated by 70% MeOH for 48 h before being injected three times with a 20 μL sample loop, and this ran for 25 min. Shimadzu Lab solution software was used to combine the data with the detection of peaks at 510 nm, and the findings were obtained by comparing them to accessible standards. For the detection of blank peaks, the plain mobile phase was employed as a control.

### Total phenolic content

The total phenolic content (TPC) was determined from the leaf, stem, and roots of *S. carvifolia* extracts from different altitudes (2,150, 2,593, and 3,178 m) using the Folin–Ciocalteu method (52). Briefly, 1 mL of extract (100–500 μg/mL) solution was mixed with 2.5 mL of 10% (w/v) Folin–Ciocalteu reagent. After 5 min, 2.0 mL of Na_2_CO_3_ (75%) was subsequently added to the mixture and incubated at 50°C for 10 min with intermittent agitation. Afterwards, the sample was cooled, and the absorbance was measured utilizing a UV spectrophotometer (Shimazu, UV-1800) at 765 nm against a blank without extract. The outcome data were expressed as mg/g of gallic acid equivalents in milligrams per gram (mg GAE/g) of dry extract.

### Total flavonoid content

The flavonoid contents of the leaf, stem, and roots of *S. carvifolia* extracts from different altitudes (2,150; 2,593; 3,178 m) were measured as per the aluminum chloride (AlCl3) assay (colorimetry). An aliquot of 1 mL of extract solution (25–200 μg/mL) or rutin (25–200 μg/mL) was mixed with 0.2 mL of 10% (w/v) AlCl_3_ solution in methanol, 0.2 mL (1 M) potassium acetate, and 5.6 mL distilled water. The mixture was incubated for 30 min at room temperature followed by the measurement of absorbance at 415 nm against the blank. The outcome data were expressed as mg/g of quercetin equivalents in milligrams per gram (mg RE/g) of dry extract (53, 54).

### Statistical analysis

The data were reported as the mean ± standard deviation. The linear regression coefficient (R^2^) for phenolic and flavonoid content with antioxidant activity was analyzed by Graph Pad Prism for Windows, Version 7 (Graph Pad Software, San Diego, CA, United States).

A linear regression analysis was used to obtain the IC50 values.

### DPPH free radical scavenging assay

The free radical scavenging ability of the leaf stem and root extracts of *S. carvifolia* from different altitudes were tested by a DPPH radical scavenging assay (55). The hydrogen atom-donating ability of the plant extractives was determined by the decolorization of the methanol solution of 2,2-diphenyl-1-picrylhydrazyl (DPPH). DPPH produces violet/purple color in methanol solution and fades to shades of yellow color in the presence of antioxidants. A solution of 0.1 mM DPPH in methanol was prepared, and 2.4 mL of this solution was mixed with 1.6 mL of extract in methanol at different concentrations (12.5–150 μg/mL). The reaction mixture was vortexed thoroughly and left in the dark at RT for 30 min. The absorbance of the mixture was measured spectrophotometrically at 517 nm. Ascorbic acid was used as a reference. The percentage DPPH radical scavenging activity was calculated by the following equation:

$$\begin{array}{c} \mathrm{Free radical scavenging activity}\;\left(\%\right)=\left(\mathrm{A0}\right)\;\mathrm{control}-\left(\mathrm{A1}\right)\;\mathrm{sample}\\ /\left(\mathrm{A0}\right)\;\mathrm{control}*100 \end{array}$$

where A0 is the control reaction absorbance, and A1 is the testing specimen absorbance. IC50 was determined graphically from the graph plotting the inhibition percentage (%) against the extracts (56).

### Sugar-reducing capacity assay

The reducing sugar content (RSC) was determined using the 3,5-dinitrosalicylic acid (DNSA) method. The measurement was performed according to the procedure of Krivorotova and Sereikaite with slight modifications (57).

The DNSA reagent was prepared by dissolving 1 g of DNSA and 30 g of sodium–potassium tartaric acid in 80 mL of 0.5 N NaOH at 45° C. After dissolution, the solution was cooled down to room temperature and diluted to 100 mL with the help of distilled water. For the measurement, 2 mL of DNSA reagent was pipetted into a test tube containing 1 mL of plant extract (1 mg/mL) and kept at 95° C for 5 min. After cooling, 7 mL of distilled water was added to the solution, and the absorbance of the resulting solution was measured at 540 nm using a UV-VIS spectrophotometer (Shimadzu UV-1800). The reducing sugar content was calculated from the calibration curve of standard D-glucose (200–1,000 mg/L), and the results were expressed as mg D-glucose equivalent (GE) per gram of dry extract weight.

### Antibacterial assay

#### Sample preparation and extraction

After cleaning, the different plant parts of the *S. carvifolia* plants collected from three altitudes (2,150, 2,593, and 3,178 m) were cut into small pieces using scissors and stored for drying. Drying was performed in a room for about 2 weeks in shade. They were then powdered using a grinder to enhance the surface area for a better extraction process. Twenty grams of finely ground powder from each plant part was used for making extracts. The 70% methanolic and aqueous extracts were prepared using a Soxhlet apparatus and simple maceration, and then the extracts were preserved for further studies.

### Antibacterial activity

The antibacterial activity of the *S. carvifolia* leaf, stem, and root extracts from three different altitudes (2,150, 2,593, and 3,178 m) was evaluated against eight strains [3 Gram +ve *Staphylococcus*: *Staphylococcus aureus* (MTCC 96), *Staphylococcus epidermidis* (MTCC 435), and *Streptococcus mutans* (MTCC 890); 5 Gram –ve: *Klebsiella pneumoniae* (MTCC 109), *Escherichia coli* (MTCC 723), *Escherichia coli* (DH5α), *Salmonella typhimurium* (MTCC 98), and *Pseudomonas aeruginosa* (MTCC741); obtained from the Microbial Type Culture Collection Centre (MTCC), Institute of Microbial Technology (IMT), Chandigarh, India] by a micro-dilution broth assay using 96-well flat bottom microtiter plates according to the CLSI guidelines, according to which the antibiotic norfloxacin was used as a standard drug, and DMSO was used as a negative control (58).

### Antifungal activity

By using the agar well diffusion method, the antifungal activity of all the *S. carvifolia* leaf, stem, and root extracts collected at different altitudes was investigated against four fungal strains: *Candida albicans* (CA-3010), *Candida albicans* (CA-227), *Creptococcus neoformans* (CN), and *Sporothrix schenckii* (SS) (59). All the fungal isolates were grown on a potato dextrose agar (PDA) at 28°C for proper sporulation. Subsequently, the fungal spores were harvested in sterile distilled water and evenly spread on PDA plates using a sterile glass spreader. Using a sterile cork borer (diameter 5 mm), wells were drilled into the agar media and filled with a sufficient amount of plant extract, oil, and water (control). The plates were allowed to rest at room temperature for 1 h to allow the extract to properly diffuse into the media before being incubated at 28°C for 96 h. The zones of inhibition around the wells were measured and recorded after incubation. The antifungal screening was performed in triplicates.

### Cytotoxicity assay

#### Sample preparation and extraction

*Selinum carvifolia* leaves, stems, and roots were collected from three altitudes and air-dried for 10 days at room temperature before being milled into a powder. Following this, 70% methanol was used to extract the dry powder for roughly 7 days at room temperature. After evaporating the solvent, dry methanolic extracts were produced. To obtain suitable solutions for the extracts, dry methanolic extracts were dissolved in dimethyl sulfoxide (DMSO).

### Cell culture

The study’s A549 cell line (adenocarcinoma human alveolar basal epithelial cells) was obtained from the American Type Culture Collection (ATCC, Manassas, Virginia, United States). The cells were grown in Dulbecco’s modified Eagle medium (Sigma) supplemented with 10% FBS (fetal bovine albumin) and 1% antibiotic/antimycin cocktail at 37°C in humidified air containing 5% CO2. The cells were allowed to develop to 80–90% confluence before being harvested with 0.25% trypsin/EDTA solution, and they were sub-cultured in 96-well plates according to the experiment’s instructions.

### Cytotoxicity assay

With minor changes (in concentration), the cytotoxicity assay based on MTT (3-(4, 5-dimethylthiazol-2-yl)-2, 5-diphenyl tetrazolium bromide) established by Mosman in 1983 was employed. A 5 mg/mL fresh stock solution of MTT was prepared in phosphate-buffered saline (PBS, pH 7.2) and filtered prior to initiating the experiment. Briefly, A549 cells (1 × 10^4^ cells/well) were seeded in a 96-well flat-bottom cell culture plate and cultured overnight (60). Furthermore, the cells were then treated with ethanolic extract of the plant at five different concentrations (1, 0.5, 0.25, 0.125, and 0.0625 mg/mL), as previously described (61). As a control, the cells were cultured in regular media (without extract and same culture conditions). After the treatment period was completed, 10 μL of MTT stock solution (1/10th volume of the total media in the well) was added to each well and incubated for 3 h under standard culture conditions. After the incubation period, the medium was withdrawn, and each experimental well was filled with 200 μL of DMSO (Sigma Aldrich), which was then incubated for 20 min at room temperature. Finally, MTT activity was measured using an ELISA plate reader set at 492 nm, and the absorbance intensity was recorded at a 550 mm wavelength (Biotek, PowerwaveXS2). The following formula was used to compute the percentage of growth inhibition:

%Cell inhibition=100−[(At−Ab)/(Ac−Ab)]×100

where At represents the absorbance value of the test substance, Ab represents the absorbance value of the blank, and Ac represents the absorbance value of the control.

IC50 values were used to express the effects of the extracts (the drug concentration reducing the absorbance of treated cells by 50% for untreated cells).

## Results

### HPLC analysis

To obtain an accurate quantification of chemicals, a sufficient resolution is required. The selection of the column, mobile phase composition, gradient flow parameters, and temperature was carried out by HPLC in order to obtain chromatograms with baseline separation of the 10 marker chemicals (ferulic acid, quercetin, gallic acid, rutin, chlorogenic acid, syringic acid, vanillic acid, cinnamic acid, caffeic acid, and protocatechuic acid) in *S. carvifolia*. To design a separation procedure for the isolates from the *S. carvifolia* extract solutions, a range of solvent systems were initially tested, starting with pure methanol and gradually adding the aqueous phase. The best-performing solvent system, consisting of methanol and water, was chosen for the current study. Eventually, all 10 phenolic marker chemicals were eluted in less than 40 min using a straightforward gradient approach based on water and methanol. With a fixed wavelength of 250 nm, the most effective detection was noted. The approach that was devised showed a good deal of specificity. The specificity of the developed analytical methods was determined by comparing the spectra’s peaks and retention durations. Consequently, we created a single stock solution in a standard manner before diluting it to the various concentration levels above and below the nominal amounts for each analyte. Based on their retention lengths and standard calibration curves, gallic acid, rutin, chlorogenic acid, syringic acid, vanillic acid, cinnamic acid, caffeic acid, and protocatechuic acid were identified and quantified from all plant components at all altitudes. In the herbal drug field, the standardization and characterization of herbal drugs are ongoing research interests together with formulations. With the development of contemporary chromatographic techniques, there is an increasing desire to create and develop simple, quick, practical, and affordable procedures for standardization. HPLC is a sensitive and precise tool that is frequently used for the quality assessment of plant extracts and the products that are made from them for standardizing the methanolic extract of plant leaves, stems, and roots (62, 63). The limitation of the study is that we have 10 phenolic compounds as markers, and these phenolic compounds have potent microbial and anticancerous activity; therefore, we only attempted to validate the plants that have these kinds of potent compounds that have been used for herbal drug formulations.

**Figure 1:**
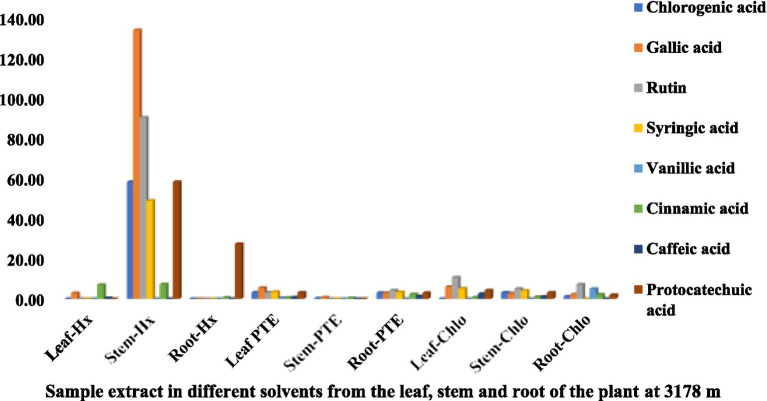
Chemical composition of leaf, stem, and root extracts [(Chl), (Hx), and (PTE)] of *Selinum carvifolia* plants (in mg/gm) growing at an altitude of 3,178 m using HPLC showing the presence of chlorogenic acid, gallic acid, rutin, syringic acid, vanillic acid, cinnamic acid, caffeic acid, and protocatechuic acid.

**Figure 2:**
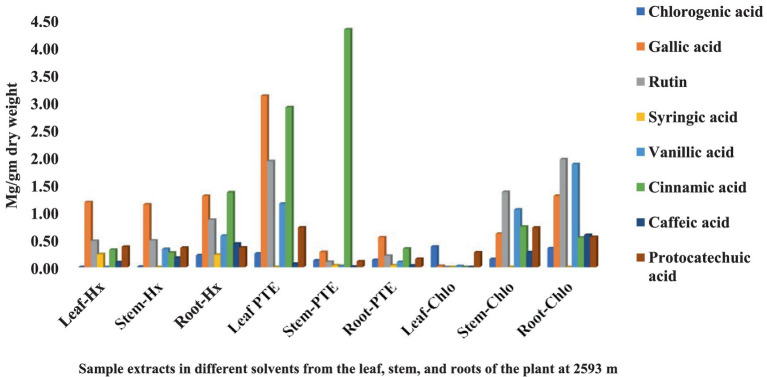
Chemical composition of leaf, stem, and root extracts [(Chl), (Hx), and (PTE)] of *Selinum carvifolia* plants (in mg/gm) growing at an altitude of 2,593 m using HPLC showing the presence of chlorogenic acid, gallic acid, rutin, syringic acid, vanillic acid, cinnamic acid, caffeic acid, and protocatechuic acid.

**Figure 3:**
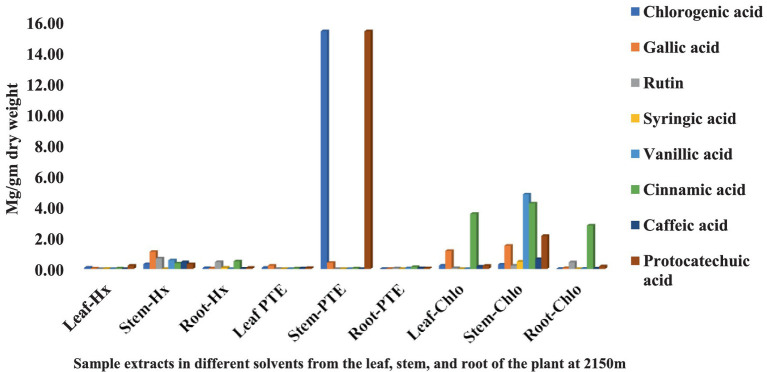
Chemical composition of leaf, stem, and root extracts [(Chl), (Hx), and (PTE)] of *Selinum carvifolia* plants (in mg/gm) growing at an altitude of 2,150 m using HPLC showing the presence of chlorogenic acid, gallic acid, rutin, syringic acid, vanillic acid, cinnamic acid, caffeic acid, and protocatechuic acid.

The HPLC fingerprints of *S. carvifolia* from three different altitudes (2,150 m, 2,593 m, 3,178 m) are given in Figures 1–3. The results of the HPLC analysis of *S. carvifolia* methanolic extract at 400 nm revealed eight different important chemicals found in various plant parts, as shown in Figure 1. Chlorogenic acid (0.25 mg/g) was identified in the smallest concentration in (PTE) stem extract, while gallic acid (13.93 mg/g) was discovered in the highest concentration in (Hx) stem extract in plants growing at an altitude of 3,178 m. Figure 2 summarizes the extract composition of different plant parts of *S. carvifolia* at an altitude of 2,593 m. The lowest concentration of chlorogenic acid (0.01 mg/gm) was discovered in the dry weight basis of (PTE) stem extract and (Hx) leaf extract, while the highest concentration of gallic acid (3.11 mg/gm) was discovered in the dry weight of (PTE) leaf. Similarly, at an altitude of 2,150 meters, Figure 3 summarizes the extract composition of plant parts of *S. carvifolia*. The results revealed that chlorogenic acid and syringic acid (0.01 mg/gm) were found in the lowest concentrations on dry weight in (PTE) and (Hx) leaf extracts, respectively, while chlorogenic acid (15.38 mg/gm) was found in the highest concentration in (PTE) stem extract and protocatechuic acid (15.12 mg/gm) in (PTE) stem extract. A single chromatogram of mixed standards and a single chromatogram of samples have been provided as Supplementary Data for reference.

### Total phenolic content

**Figure 4:**
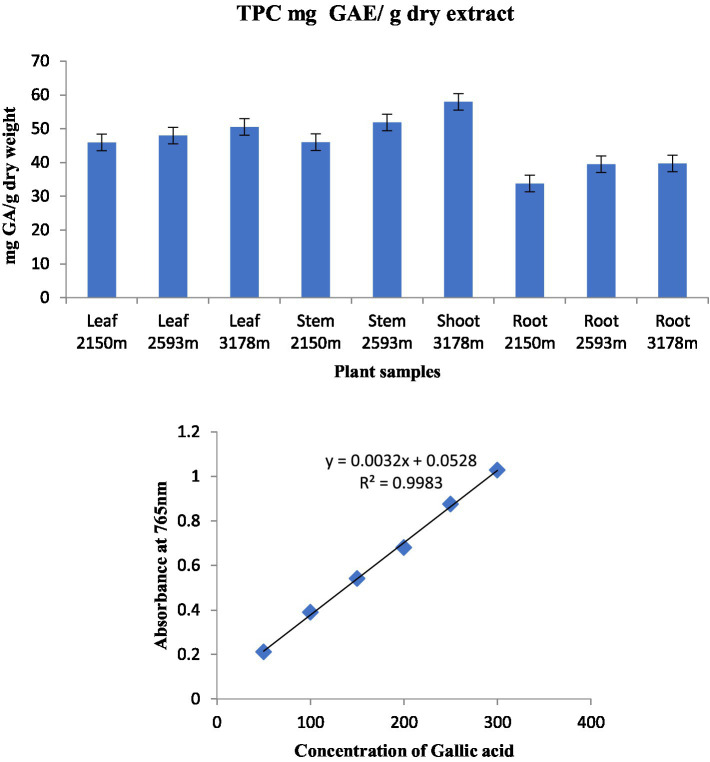
Total phenolic content in various plant components along various altitudinal gradients and a standard curve with gallic acid concentrations ranging from 0.5 to 4 g/mL.

The total phenolic content (TPC) of standard gallic acid was estimated using the calibration curve (Y = 0.0032x + 0.0528, R2 = 0.9983) and represented as mg GAE/g dry extract weight. The TPC of the investigated plant samples ranged from 33.79 to 57.95 mg GAE/g dry extract weights (Figure 4). The highest TPC level was found in stem extract at an altitude of 3,178 m, followed by stem extract at 2,593 m (57.95 and 51.85 mg GAE/g dry extract weight), while the lowest content was found in root extract at an altitude of 2,150 m (33.79 mg GAE/g dry extract weight) among the nine plant samples analyzed. This is the first study of TPC in *S. carvifolia* leaves, stems, and roots and their variation at different altitudes (2,150, 2,593, and 3,178 m).

### Total flavonoid content

**Figure 5:**
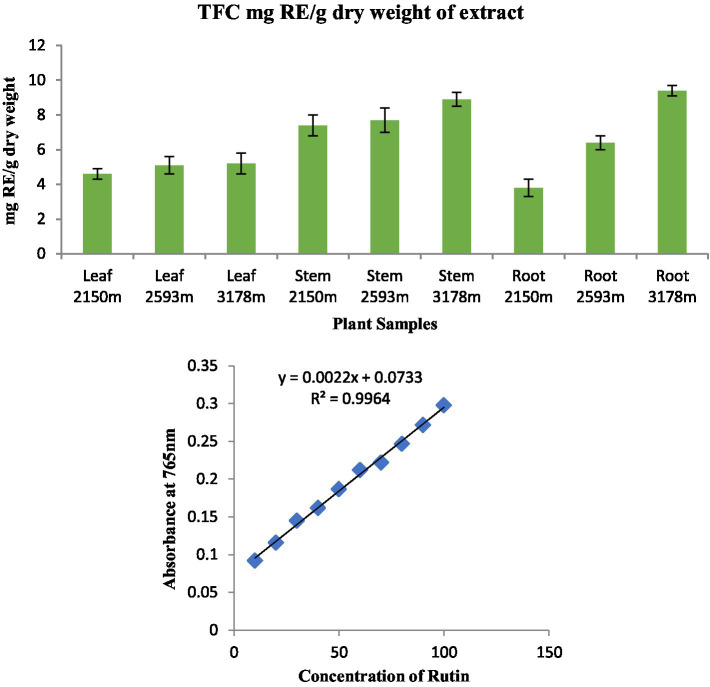
Total flavonoid content in different plant parts along various altitudinal gradients and a standard curve with rutin concentration ranging from 5 to 25 μg in the reaction mixture.

The equation obtained from the standard rutin (R) graph was used to calculate the total flavonoid content (TFC) in the organic extract of the analyzed plant parts. TFC was found in considerable amounts in all plant parts (Figure 5). The highest TFC content was found in root extract at 3,178 m (9.4 mgRE/g) and the lowest in root extract at 2,150 m (3.8 mgRE/g).

### Antioxidant activity

**Figure 6:**
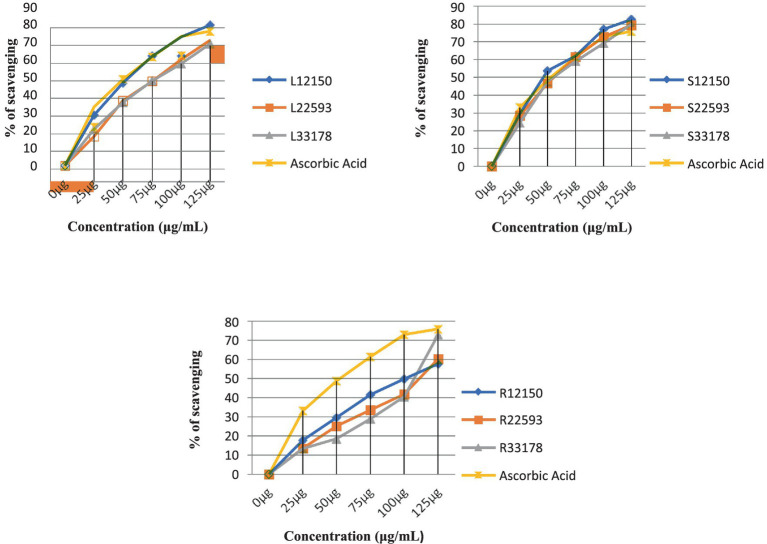
Measurement of DPPH radical scavenging activity in leaf, stem, and root extracts at various altitudes (Triple experiments were carried out). The data are presented as mean ± SD; *n* = 3, *p* < 0.05.

**Figure 7:**
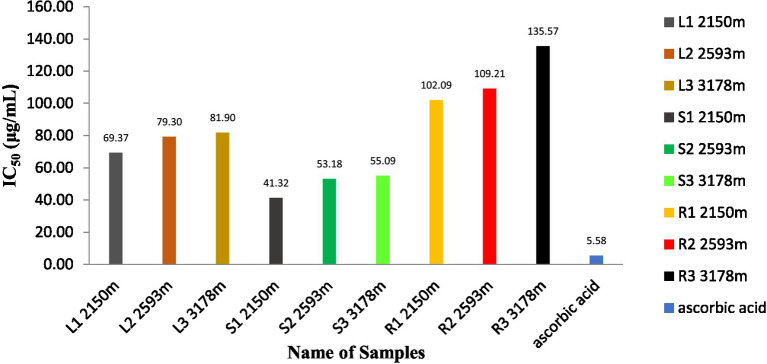
Determination of the IC50 value in leaf, stem, and root extracts at different altitudes.

As shown in Figure 6, the antioxidant activity of methanolic extracts of *S. carvifolia* plant parts collected at various altitudes was assessed using the DPPH free radical scavenging test. In a concentration-dependent way, all the extracts show promising antioxidant activity. The IC50 value of the plant extracts and ascorbic acid as a positive control was graphically derived from the graph of the DPPH inhibition percentage. We found excellent efficiency of scavenging activity in extracts of the leaves, stems, and roots of *S. carvifolia* from different altitudes (2,150, 2,593, and 3,178 m; Figure 7).

### Reducing sugar activity

The reducing sugar capacity (RSC) of different plant part extracts (leaves, stems, and roots) from different altitudes (2,150, 2,593, and 3,178 m) in *S. carvifolia* was studied using the known standard of ascorbic acid (AA), with the concentration level ranging from 250 ug/mL to 1 mg/mL. RSC was determined using the standard D-glucose calibration curve and represented as μg/mL GE/g dry extract weight. The RSC of the investigated plant samples ranged from 0.747 to 0.734 μg/mL GE/g dry extract weight. The RSC was highest in the leaf and stem extracts at an altitude of 3,178 m (0.747 and 0.734 ug/1 mL GE/g dry extract weight, respectively) among the nine plant samples examined. The lowest RPC was found in the root extract at an altitude of 2,150 m (0.495 μg/mL of GE/g dry extract).

### Antibacterial activity

The plant extracts obtained from different plant parts collected from three altitudes (2,150, 2,593, and 3,178 m) were tested for antibacterial activity on strains of *Staphylococcus aureus* (SA), *Staphylococcus epidermidis* (SE), *Streptococcus mutans* (SM), *Klebsiella pneumoniae* (KP), *Escherichia coli* (EC), *Escherichia coli* (DH5α), *Salmonella typhimurium* (STM), and *Pseudomonas aeruginosa* (PA). Root extract of the plant collected from all altitudes was alone found to be active against SA-96, while other plant parts failed to show any kind of activity against the bacterial strains listed. Methanolic extract of the root collected from an altitude of 2,150 m showed the highest activity (<250 μg/mL) on the SA-96 strain. Similar activity was also recorded from the roots collected at 2,593 m on SA-96 and moderate activity (<500 μg/mL) on KP and PA. On the other hand, extracts from roots collected at 3,178 m showed moderate activity (<500 μg/mL) on both strain SA-96 and SE strains (Table 1).

**Table 1:** Minimum inhibitory concentration of ethanolic extract of roots (in μg/mL) of *Selinum carvifolia* against *Staphylococcus aureus* (SA-96), *Staphylococcus epidermidis* (SE), *Streptococcus mutans* (SM), *Klebsiella pneumoniae* (KP), *Escherichia coli* (EC), *Escherichia coli* (DH5α), *Salmonella typhimurium* (STM), and *Pseudomonas aeruginosa* (PA).

Altitudes	Samples strains							
	SA-96	SE	EC	KP	SM	DH5α	STM	PA
Root 2,150 m	250	--	--	--	--	--	--	--
Root 2,593 m	250	--	--	<500	--	--	--	<500
Root 3,178 m	500	500	--	--	--	--	--	--

Aqueous extracts from leaves, stems, and roots from plants at three different altitudes (2,150, 2,593, and 3,178 m) were also tested for antibacterial activity against same bacterial strains. However, no activity was found against any bacterial strain except that of stem growing at an altitude of 3,178 m with moderate activity (>500 μg/mL) against PA (Table 2).

**Table 2:** Minimum inhibitory concentration of aqueous extract of stems (in μg/mL) of *Selinum carvifolia* against *Staphylococcus aureus* (SA-96), *Staphylococcus epidermidis* (SE), *Streptococcus mutans* (SM), *Klebsiella pneumoniae* (KP), *Escherichia coli* (EC), *Escherichia coli* (DH5α), *Salmonella typhimurium* (STM), and *Pseudomonas aeruginosa* (PA).

Altitudes	Samples strains							
	SA-96	SE	EC	KP	SM	DH5α	STM	PA
Stem 2,150 m	--	--	-	-	--	--	--	--
Stem 2,593 m	--	--	--	--	--	--	--	--
Stem 3,178 m	--	--	--	--	--	--	--	<500

### Antifungal activity

Methanolic and aqueous plant part extracts (leaves, stems, and roots) collected from three different altitudes (2,150, 2,593, and 3,178 m) were tested for activity against the fungal strains *Candida albicans* (CA-3010), *Candida albicans* (CA-227), *Creptococcus neoformans* (CN), and *Sporothrix schenckii* (SS). The root extracts belonging to plants growing at altitudes of 2,593 and 3,178 m were found to be active against the SS (<500 μg/mL). The activity (<500 μg/mL) against CA-227 was also reported in the extract of plant collected at an altitude of 3,178 m. However, no significant activity was reported against any fungal strains in the extract from plants growing at 2,150 m altitude (Table 3). Similar activity was also recorded in the aqueous extracts of roots growing at altitudes of 2,593 and 3,178 m. On the other hand, activity (500 μg/mL) against the SS fungal strain was recorded in the aqueous extract of roots growing at 2,593 and 3,178 m (Table 4).

**Table 3:** Minimum fungicidal concentration of ethanolic extract of roots (in μg/mL) of *Selinum carvifolia* against *Candida albicans* (CA-3010), *Candida albicans* (CA-227), *Cryptococcus neoformans* (CN), and *Sporothrix schenckii* (SS).

Altitudes	Samples strains			
	CA-3010	CN	SS	CA-227
Root 2,150 m	--	--	--	--
Root 2,593 m	--	--	<500	--
Root 3,178 m	--	--	<500	<500

**Table 4:** Minimum fungicidal concentration of aqueous extract of roots (in μg/mL) of *Selinum carvifolia* against *Candida albicans* (CA-3010), *Candida albicans* (CA-227), *Cryptococcus neoformans* (CN), and *Sporothrix schenckii* (SS).

Altitudes	Samples strains			
	CA-3010	CN	SS	CA-227
Root 2,150 m	--	--	--	--
Root 2,593 m	--	--	<500	--
Root 3,178 m	--	--	<500	--

### Cytotoxicity

**Figure 8:**
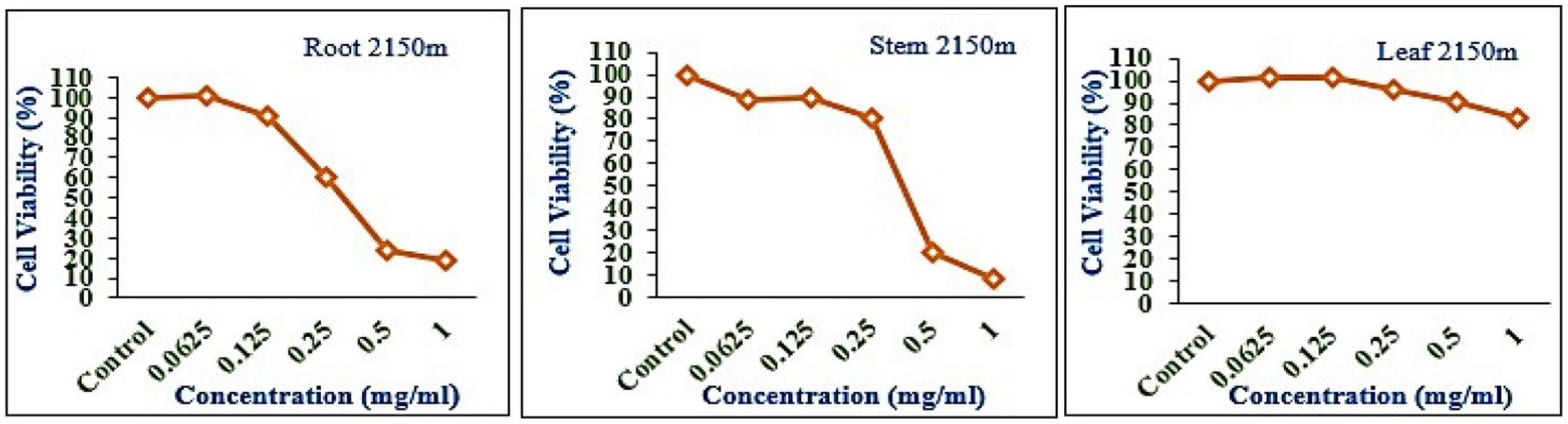
Inhibition of A549 cell proliferation in methanolic extract of different plant parts (root, stem, and leaf) of *Selinum carvifolia* at 2,150 m altitude.

**Figure 9:**
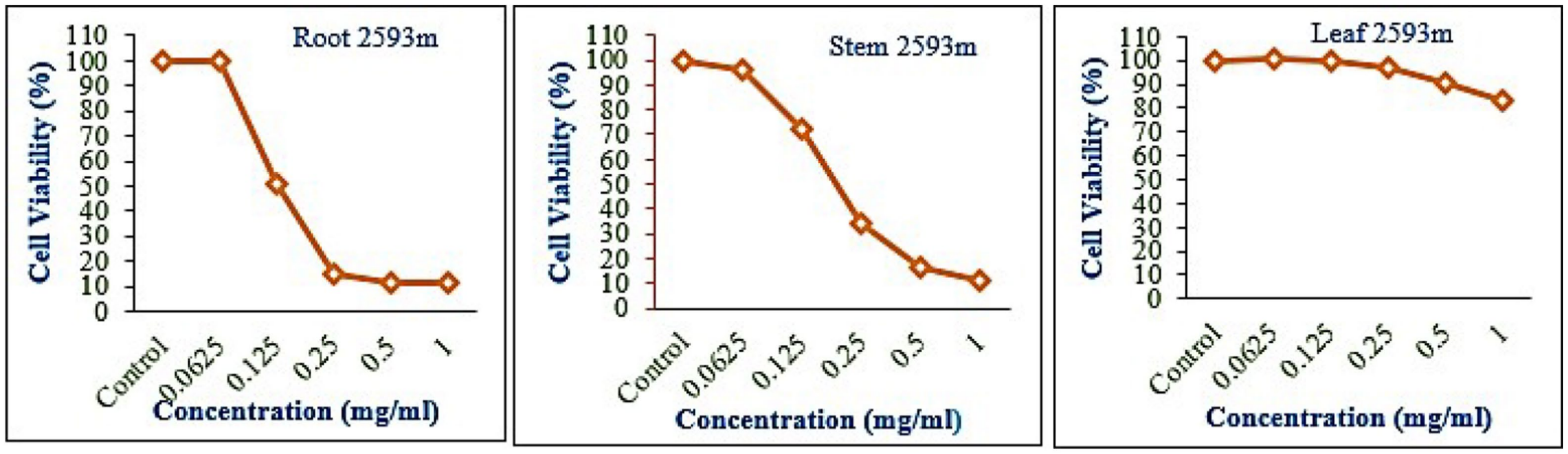
Inhibition of A549 cell proliferation in methanolic extract of different plant parts (root, stem, and leaf) of *Selinum carvifolia* at 2,593 m altitude.

**Figure 10:**
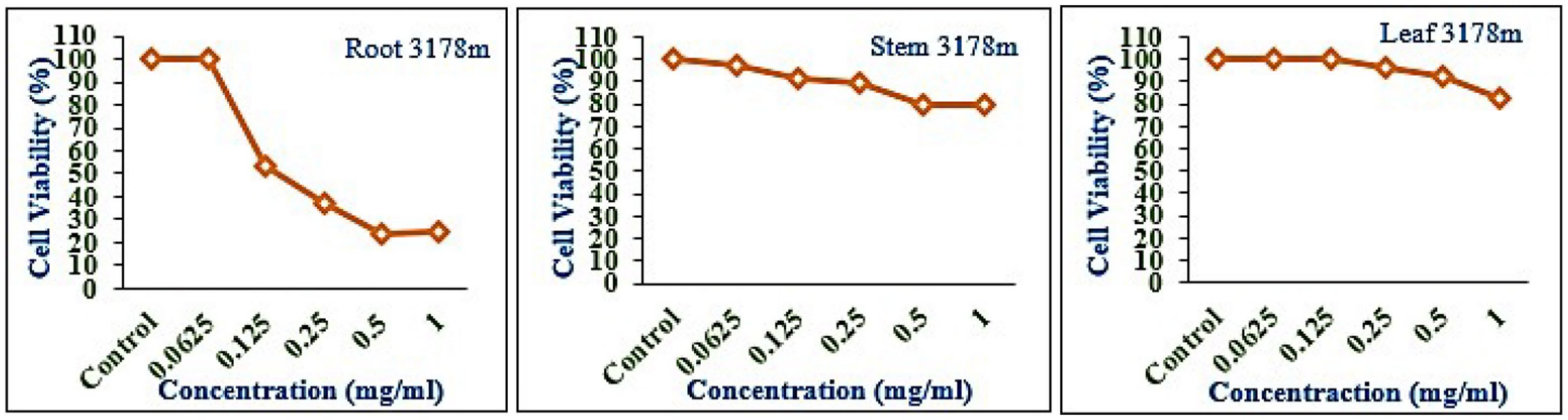
Inhibition of A549 cell proliferation in methanolic extract of different plant parts (root, stem, and leaf) of *Selinum carvifolia* at 31,78 m altitude.

**Figure 11:**
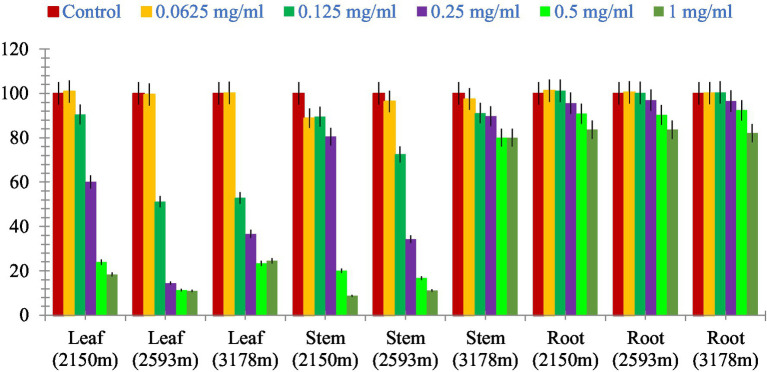
Comparative graphical representation of inhibitory activity of plant part extracts (leaf, stem, and roots) from three different altitudes (2,150, 2,593, and 3,178 m) against A549 cell proliferation using MTT assay. Data are represented as mean ± SE value of the three independent experiments.

The leaf, stem, and root extracts of plants growing at different altitudes were tested against A549, and it was observed that leaf extract does not have any cytotoxic activity even at higher doses. However, the stem and root extracts showed an inhibitory effect at a 0.25 mg/mL concentration, while complete cell cytotoxicity was found at the highest dose of (1 mg/mL) concentration. *Selinum carvifolia* leaf extracts from different altitudes showed mild cytotoxicity or inhibition of cell proliferation in higher concentrations, while root extracts of plants growing at the three altitudes (2,150, 2,593, and 3,178 m) inhibited cell proliferation even at a lower concentration of 0.125 mg/mL, and maximum cell growth inhibition could be seen at higher concentrations of 1 mg/mL. Plants growing at an altitude of 2,150 m displayed modest cytotoxicity at higher dosages, but plants growing at altitudes of 2,593 and 3,178 m exhibited cytotoxicity in A549 cells at lower concentrations, resulting in full cell death in a dose-dependent manner. The mild toxicity of leaf extracts from plants growing at the three altitudes (2,150, 2,593, and 3,178 m) and the stem of plants growing at an altitude of 3,178 m was detected, and the percentage of growth inhibition increased with the increasing concentration of test chemicals (as demonstrated in the graphs in Figures 8–10). A comparative graphical representation of the inhibitory activity of extracts from different plant parts (leaves, stems, and roots) of *S. carvifolia* collected from three altitudes (2,150, 2,593, and 3,178 m) against the A549 cell line using an MTT assay is shown in Figure 11.

## Discussion

Phytochemical analysis is a significant laboratory or scientific process. This procedure identifies the fundamental elements of any plant portion, including the leaves, stems, and roots. Nobody is exactly sure how many distinct types of medicinal plants are utilized globally today, but we do know that they play a significant role in both conventional and Western medicine. Thus, it is crucial to use a viable technique to test the phytochemicals present in the plant. It can be inferred from the HPLC fingerprints that this analytical method is a practical way to determine the presence of a wide range of substances contained in the methanolic extract of plant leaves, stems, and roots.

Various medicinal plants are used traditionally for the treatment of different diseases or symptoms. In order to correlate the traditional uses to modern pharmacological activities, it is important to analyze their major chemical constituents and to evaluate their biological activities. To gain a broad picture of a plants’ phytochemical composition and its biological activities, it is essential to investigate different portions of the plant (leaf, stem, and root bark, fruits, flowers, and so on) to find the most promising source (64). Using reversed-phase HPLC with a UV detector at 230-400 nm, we investigated several components of *S. carvifolia* (root, stem, and leaf) and discovered that chloroform, hexane, and petroleum ether extracts contain substantial levels of phenolic compounds. The phenolic chemicals in the leaves, stems, and roots of *S. carvifolia* collected from three different altitudes were elucidated. Based on their retention lengths and standard calibration curves, gallic acid, rutin, chlorogenic acid, syringic acid, vanillic acid, cinnamic acid, caffeic acid, and protocatechuic acid were identified and quantified from all the plant parts at each studied altitude. It was found that the concentration of phenolic compounds increased significantly along with the altitude in both aerial and underground parts. The leaves and stem of all three different altitudes contained more phenolic compounds than roots. The presence of physiologically and pharmacologically essential phenolic chemicals in quantifiable amounts as demonstrated in the study can be used by the pharmaceutical and phytopharmaceutical industries to build quality control profiles. Furthermore, this method used is accurate and repeatable, and it may be used to determine phenolic compounds in plant extracts.

To the best of our knowledge, this study is the first to isolate, characterize, biologically evaluate, quantify, and validate the chemical contents of *S. carvifolia* using HPLC. The results of this study reveal the significant *in vitro* cytotoxic potential of this plant toward cancerous cells, which suggests the anticancerous activity of plant extract can probably be attributed to the phenolic compounds, as determined by the MTT cytotoxicity assay. However, more studies are warranted to further elucidate the properties of the compounds present in the extracts and their mechanism of action. The presence of highly bioactive molecules creates new opportunities for therapeutic development. The created method is easy to use, sensitive, specific, and repeatable, and it can be expanded to assess the quality of *S. carvifolia*, according to the validation results. For quick study of its phytomolecules in diverse plants, herbal formulations, and plant products, the preliminary HPLC fingerprinting approach can be useful.

Susceptibility tests with MICs in the range of 500–1,500 g/mL are commonly used to classify the antimicrobial activity of plant extracts (65). In our findings, antibacterial activity of methanolic extract in the roots of *S. carvifolia* growing at an altitude of 2,150 m was reported to be active against only one strain among the selected bacterial strains, namely, SA-96, while the root parts collected from an altitude of 2,593 m showed activity against SA-96, KP, and PA strains. The methanolic extracts of roots collected from an altitude of 3,178 m showed moderate activity against SA-96 and SE strains, whereas the aqueous extracts of stems from the same altitude showed activity against the PA strain. The root methanolic extract from an altitude of 3,178 m showed antifungal activity against two out of four fungal strains, namely, SS and CA-227, while the roots obtained from an altitude of 2,593 m only showed activity against the SS strain. Antifungal activity was found in the aqueous extracts of roots from the altitudes of 2,593 and 3,178 m against the SS strain. This variation could be attributed to differences in the active compounds present in the extracts, extraction solvent, employed plant component, analysis method, environmental stress, and climatic and geographical factors (66).

The antioxidant properties of *S. carvifolia* were assessed by measuring total phenols, total flavonoids, and DPPH activity using the crude methanolic extract of various plant parts from different altitudes, and it was observed that there were variations in activity in the different altitudes, which could be due to several factors. Reducing sugar activity was found to be at a maximum in the methanolic extracts of the aerial parts as the altitude increased. The genotype, plant age, soil quality, geographical location, meteorological conditions, cultivation method, and abiotic stress may all play a role in this variation (67–69).

The cytotoxicity test was performed on the A549 cell line to determine whether the fractions were cytotoxic to cancer cell lines. The cytotoxic data of different fractions of the extracts of *S. carvifolia* revealed no toxicity in the extracts of the aerial parts, even at different altitudes from the stems collected at 3,178 m, but the root extracts showed mild inhibition at high doses. These results confirm the beneficial use and application of this plant against human pathogens and the associated human-and community-acquired infections (70, 71).

In the present investigation, it was interesting to observe that the total phenolic contents in nearly all the plant parts increased with altitude, thus also increasing the antibacterial, antifungal, and antioxidant properties along with the cancer cell cytotoxicity and reducing sugar activities. The secondary metabolite concentration increases with altitude as it plays an important role in the defense mechanisms of plants. The magnified UV radiation and low temperature produces a stressful environment, leading to the formation of free radicals that are countered by phenols, flavonoids, and terpenoids (72). Among the different plant parts tested, the bioactivity was found to be highest in the roots, which could be an indication about the importance of roots for the adaptation of plants to a changing environment, even at the lowest level, as also supported by Souhir et al. (73). The cytotoxic activity of plant has also been shown to increase with the increase in altitude, with better activity observed against cancer cell lines (74).

Our findings are intriguing, and they may prompt additional investigation into the phytochemical, toxicological, and pharmacological characteristics of these extract products in order to promote their widespread use in antibacterial, antifungal, antioxidant, and anticancer activities. In the current study, the methanolic extracts gave higher yields of chemical constituents than initially expected or anticipated; the originality of this work is that positive results were achieved with a hydro-alcohol ratio and it will be useful to carry out other data analyses with MIC and other formulation studies, because hydro-alcohol is more suitable for clinical study than methanol or water extracts. In comparison to standard drugs, hydro-alcoholic extracts of *S. carvifolia* were found to be active against the majority of clinically isolated microorganisms and fungi. The current study validated the claimed uses of the whole plant in traditional medicine to treat various infectious diseases caused by microbes. However, more research is needed to better assess the potential efficacy of crude extracts as antimicrobial agents. The altitudinal variation was also found in the plant extract, and according to our findings, the plant extract growing at the highest altitude produces the best results.

## Conclusion

Biologically active compounds were identified and quantified in the leaf stems and roots of *Selinum carvifolia*. Eight phenolic compounds were analyzed through HPLC methods. For the first time, the presence of gallic acid, chlorogenic acid, syringic acid, vanillic acid, cinnamic acid, caffeic acid, and protocatechuic acid have been reported as non-volatile constituents of *S. carvifolia*. A significant difference in concentration was observed in phenolic compounds from lower to higher altitudes. This may be due to the fact that at higher altitudes, plants encounter greater environmental challenges than in lower altitudes. All the extracts showed antibacterial, antifungal, cytotoxicity, and antioxidant effects, notably the root extracts, which exhibited very good cytotoxicity activity and scavenging activity against DPPH radicals. *S. carvifolia,* which was researched in this study, is a rich source of physiologically active chemicals that can be utilized to treat and prevent a variety of ailments.

In the current study, the methanolic extract gave higher yields of chemical constituents than expected; the originality of this work is that some great results were obtained with the hydro-alcohol ratio, and it will be useful to carry out other data analyses with MIC and other formulation studies, because hydro-alcohol is more suitable for clinical study than methanol or water extracts. In comparison to standard drugs, the hydro-alcoholic extracts of *S. carvifolia* were found to be active against the majority of clinically isolated microorganisms and fungi. The current study validated the claimed uses of the whole plant in traditional medicine to treat various infectious diseases caused by microbes. However, more research is needed to better assess the potential efficacy of crude extracts as antimicrobial agents. The altitudinal variation was also found in the plant extract, and according to our findings, the plant extract growing at the highest altitude produced the best results. The current findings will be used to select plant species for further investigation in the search for new natural bioactive compounds.

The phytochemical composition, total phenol content, and antibacterial, antifungal, cytotoxicity, and DPPH activity of the methanolic extracts of *S. carvifolia* leaves, stems, and roots were investigated in this study. The antioxidant activity of different parts of *S. carvifolia* MeOH extract was tested against free radicals DPPH and reducing sugar activity. The results showed that root extract is a good antioxidant agent because it inhibits DPPH activity more effectively. The differences in the antioxidant activity of the different parts of *S. carvifolia* can be attributed to differences in the concentration of the identified phenolic compounds present and the higher levels of phenolics and flavonoids in the extract of the selected samples.

According to the current findings, this plant is a rich source of medicinally important phytoconstituents, and the observed antimicrobial, cytotoxic, and antioxidant potential could be attributed to these constituents. Although the parameters used in this study are not disease-specific, quantification of their properties can be used to guide the use of these plants in ROS-related complications such as diabetes. More research is needed to isolate and identify the responsible active compounds and their mechanisms of action so that we can better understand their ability to control relevant diseases.

## Data availability statement

The raw data supporting the conclusions of this article will be made available by the authors, without undue reservation.

## Author contributions

The manuscript was structured and prepared by RS, LS, NS, SP, AS, HD, PV, SS, SK, SM, and SR under the guidance of GS. All authors contributed to the article and approved the submitted version.

## Conflict of interest

The authors declare that the research was conducted in the absence of any commercial or financial relationships that could be construed as a potential conflict of interest.

## Publisher’s note

All claims expressed in this article are solely those of the authors and do not necessarily represent those of their affiliated organizations, or those of the publisher, the editors and the reviewers. Any product that may be evaluated in this article, or claim that may be made by its manufacturer, is not guaranteed or endorsed by the publisher.

## Figures and Tables

**Table:** 

HPLC	High-pressure liquid chromatography
DNSA	3,5-dinitrosalicylic acid
MIC	Minimum inhibitory concentrations
DPPH	2,2-diphenyl-1-picrylhydrazyl
MTT	(3-(4,5-dimethylthiazol-2-yl)-2,5-diphenyltetrazolium bromide)
A549	Adenocarcinomic human alveolar basal epithelial cells
NBRI	National Botanical Research Institute
GPS	Global positioning system
GBPIHED	Govind Ballabh Pant Institute of Himalayan Environment and Development
Hx	Hexane
Chl	Chloroform
PTE	Petroleum ether
TPC	Total phenolic content
TFC	Total flavonoid content
GA	Gallic acid
MTCC	Microbial type culture collection and gene bank
CLSI	Clinical and Laboratory Standards Institute
DMSO	Dimethyl sulfoxide
PDA	Potato
Dextrose	Agar
ATCC	American type culture collection
ELISA	Enzyme-linked immuno-sorbent assay
EDTA	Ethylene diamine tetra-acetic acid
